# Association between season of conception, month of conception with preterm birth in China: a population-based retrospective cohort study

**DOI:** 10.1186/s12978-023-01636-6

**Published:** 2023-06-13

**Authors:** Yu Wu, Hanfeng Ye, Yanling Yuan, Cai Kong, Wenzhan Jing, Jue Liu, Min Liu

**Affiliations:** 1 Department of Epidemiology and Biostatics, School of Public Health, Peking University, No. 38, Xueyuan Road, Haidian District, Beijing, 100191 People’s Republic of China; 2 Yunnan Population and Family Planning Research Institute, No. 146, Qingnian Road, Wuhua District, Kunming, 650021 Yunnan China

**Keywords:** Conception season, Conception month, Preterm birth, Prevalence, China

## Abstract

**Background:**

Seasonal patterns of preterm birth were identified in previous studies, but the effect of conception season on preterm birth has not been extensively studied. Based on the notion that the etiological roots of preterm birth lie in the beginning of pregnancy, we did a population-based retrospective cohort study in Southwest China to examine the effects of season of conception and month of conception on preterm birth.

**Methods:**

We did a population-based retrospective cohort study in women (aged 18–49) who participated in the NFPHEP from 2010 to 2018, and had a singleton livebirth in southwest China. According to the time of the last menstruation reported by the participants, month of conception and season of conception were then ascertained. We used multivariate log-binomial model to adjust the potential risk factors for preterm birth and obtained adjusted risk ratio (aRR) and 95% confidence intervals (95%CI) for conception season, conception month and preterm birth.

**Results:**

Among 194 028 participants, 15 034 women had preterm birth. Compared with pregnancies that were conceived in the summer, pregnancies that were conceived in the spring, autumn and winter had the higher risk of preterm birth (Spring: aRR = 1.10, 95% CI: 1.04–1.15; Autumn: aRR = 1.14, 95% CI: 1.09–1.20; Winter: aRR = 1.28, 95% CI: 1.22–1.34) and also had a higher risk of early preterm birth (Spring: aRR = 1.09, 95% CI: 1.01–1.18; Autumn: aRR = 1.09, 95% CI: 1.01–1.19; Winter: aRR = 1.16, 95% CI: 1.08–1.25). Pregnancies in December, and January had a higher risk of preterm birth and early preterm birth than pregnancies that were conceived in July.

**Conclusions:**

Our study found that preterm birth was significantly related to season of conception. Preterm and early preterm birth rates were the highest among pregnancies that were conceived in winter, and the lowest among pregnancies in summer.

## Introduction

Preterm birth is a global public health problem [1]. Approximately 944 thousand neonates died from preterm birth, accounting for 35.3% of all neonatal deaths [2, 3]. Babies born before 34 weeks of gestation (early preterm birth) have only a 50% chance of survival [4]. In addition, preterm birth may increase risks of neonatal respiratory diseases, necrotizing enterocolitis, sepsis, neurological conditions and et al. [5]. In the long term, preterm birth has been linked to behavioral, social-emotional, and learning difficulties in childhood [6], which causes psychological and economic burdens for the families of preterm neonates. Preterm birth has long been known to be a complex clinical syndrome with multiple etiologies and presentations [7]. The pathological processes implicated in the preterm birth syndrome include intrauterine infection, uterine ischemia, uterine over-distension, abnormal allogenic recognition, allergic-like reaction, cervical disease, and endocrine disorders [8]. In addition, many risk factors are related to preterm birth, including race, maternal age, lower socioeconomic status [7], multiple pregnancy [9], shorter pregnancy intervals [9–11], smoking during pregnancy [9], genetic factors [12], etc.

Seasonal patterns of preterm birth have been found in previous studies [13]. An early study in the United States found that autumn was the peak season for preterm birth, with the highest preterm birth rate in September and the lowest preterm birth rate in May [14]. The study by Lee et al. [15] in the United Kingdom, the study by Matsuda et al. [16] in Japan, and the study by Baroutis et al. [17] in Greece all found that preterm birth rate showed an annual pattern with two peaks (Winter and Summer). This may be due to some climatic factors such as ambient temperature, humidity, the length of daylight and atmospheric pressure [18]. In addition, season-related living habits, religious affections (Muslims are required to fast during the month of Ramadan [19]), social customs (time of holidays, seasonal marriage), and economic and cultural factors [17] may also affect preterm birth. An exploration of seasonality of preterm birth may provide a direction in the search for risk factors.

Currently, the effect of conception season on preterm birth has not been extensively studied. As maternal diet, exposure to infectious agents, and ambient temperature vary throughout the year, season of conception can serve as a proxy for these factors. Geographical, cultural and socio-economic differences in different countries or regions may affect the association between season of conception and preterm birth.

China is the country with the second largest number of preterm births in the world [1], so addressing preterm birth is critical to reduce neonatal and child under 5 years morbidity. The cause of preterm birth was unclear, and we focused on season of conception based on the notion that the etiological roots of spontaneous preterm birth lie in the beginning of pregnancy. We did a large population-based retrospective cohort study in women of childbearing age in Southwest China to examine the influence of conception season and conception month on preterm birth.

## Materials and methods

### Data sources

We conducted a population-based retrospective cohort study involving women aged 18–49 years who participated in the National Free Preconception Health Examination Project (NFPHEP) from Jan 1, 2010, to Dec 31, 2018, and had delivered a singleton livebirth in 129 counties in southwest China. NFPHEP was a project initiated by the Chinese National Health and Family Planning Commission in 2010, and integrated into China’s National Basic Public Health Service Program in 2019. It aims to provide free health examinations and other services before conception for couples who planned to become pregnant in the next 6 months, provide pre-pregnancy check-up, early pregnancy follow-up and post-delivery follow-up to women of childbearing age. This study was approved by the Institutional Review Board of the Chinese Association of Maternal and Child Health Studies (AMCHS-2014-6). All participants provided written informed consent before enrolment [20].

After a survey of the fertility desire of women of childbearing age by local health workers, all women who wish to be pregnant in the next 6 months were recruited and given free medical examinations. A standardized questionnaire was used to collect baseline information from women by trained qualified local health workers, including demographic characteristics and reproductive characteristics. The height, weight and body mass index (BMI) of the participants were obtained from physical examination data. Hemoglobin concentrations and hepatitis B serological markers were obtained from laboratory tests. In this study, the diagnostic criteria for anemia referred to the diagnostic threshold recommended by World Health Organization (anemia was defined as lower than 120 g/L for non-pregnant women) and adjusted according to altitude [21].

In the first trimester of the participants, the maternal and child health workers interviewed participants face-to-face or by telephone and investigated whether participants ate meat and eggs, ate vegetables, smoked, consumed alcohol, and took folic acid during pregnancy.

All participants were followed up by maternal and child health workers for 1 month after delivery to collect information including pregnancy outcome (normal birth, preterm birth, abortion, stillbirth or induced labor), delivery date, gestational weeks, and newborn information (singleton or multiple births). The study was terminated when participants had preterm birth or other pregnancy outcomes, or when the study reached the end of the observation period (December 31, 2018).

## Study population

**Fig. 1:**
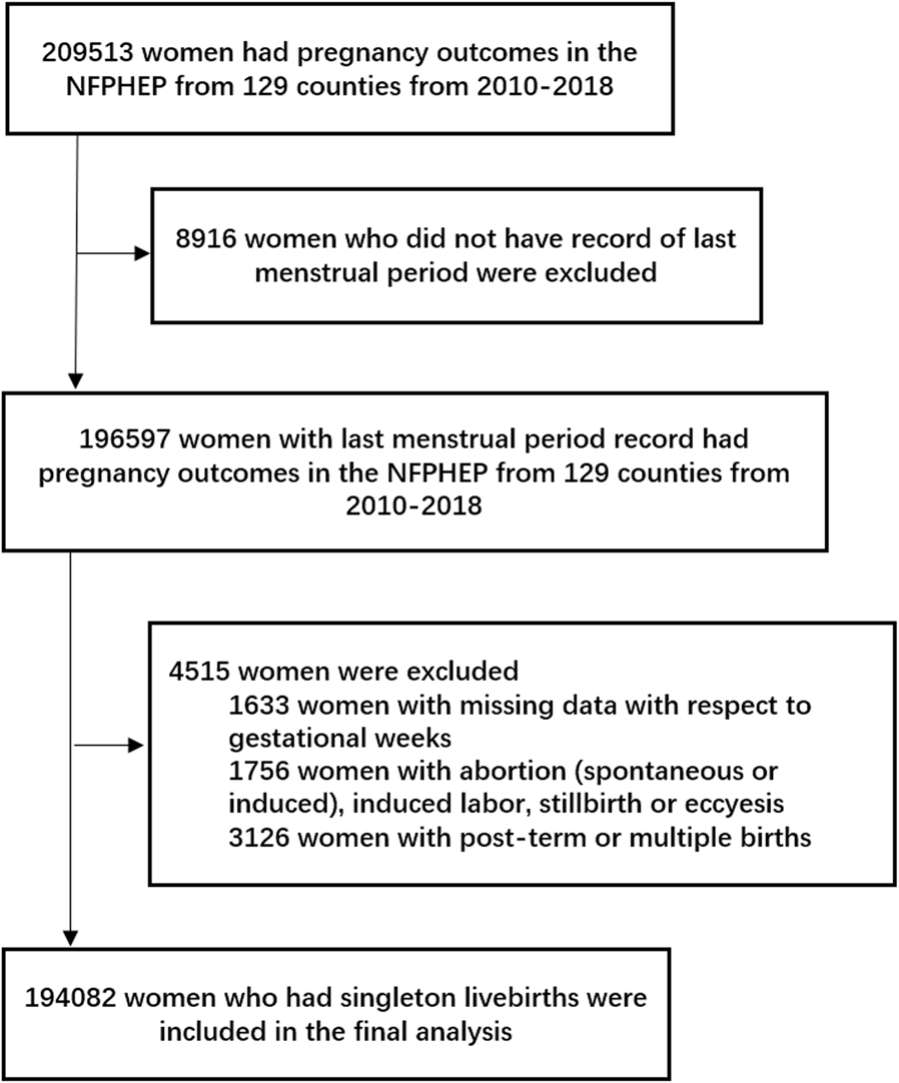
Inclusion and exclusion criteria of participants

In this study, women of childbearing age 18–49 years who participated in NFPHEP from January 1, 2010 to December 31, 2018 in 129 counties in Southwest China and had childbirth outcomes were selected as the research subjects. The selection process of the subjects was shown in Fig. 1.

Inclusion criteria:

1. Women of childbearing age who voluntarily participated in NFPHEP with informed consent between January 1, 2010 and December 31, 2018;
2. Aged 18–49 years old;
3. Had the outcome of childbirth.

Exclusion criteria:

1. Missing the time record of last menstrual period;
2. Missing the gestational week of childbirth;
3. Miscarriage, stillbirth and labor induction after pregnancy;
4. Post-term or multiple births.

### Ascertainment of season and month of conception

With the vast Asian continent to the north and the vast Indian Ocean and Pacific Ocean to the south, southwest China is located in subtropical northern latitude. Most of the territory has a typical temperate climate characteristic. In spring (March 21 to June 20), the average monthly temperature is mostly below 20 ℃, with little rain and a large temperature difference between day and night. In summer (June 21 to September 20), the average monthly temperature in most regions is about 22 ℃ and the precipitation is large, accounting for more than 60% of the year. In autumn (September 21 to December 20), the temperature in most areas is about 2 ℃ lower than the spring and the weather is dry with precipitation less than half of the summer precipitation. In winter (December 21 to March 20), the average monthly temperature is 6–8 ℃, sometimes with frost or snow and the precipitation is small, accounting for only 3–5% of the whole year.

In this study, according to the time of the last menstruation reported by the participants during the first trimester follow-up, month of conception was then ascertained. We used astronomical birth season in our study, based on solstices and equinoxes as the bounds of the season categories. Four astronomical birth seasons were created following the standard definition of spring (March 21 to June 20), summer (June 21 to September 20), autumn (September 21 to December 20), and winter (December 21 to March 20).

### Ascertainment of preterm birth

The primary outcome was preterm birth which was defined as a delivery from 28 weeks to less than 37 weeks of gestation. Early preterm birth was defined as a delivery from 28 weeks to less than 34 weeks of gestation. Preterm birth rate and early preterm birth rate were the proportion of preterm births and early preterm births in the total number of all singleton livebirths, respectively.

### Ascertainment of covariates

Covariates related to preterm birth and season of conception were adjusted, including demographic characteristics, reproductive characteristics, health status, and lifestyle habits during pregnancy. Demographic characteristics included age (18–20years, 21–25years, 26–30ywars, 31–35years, 36–49years), ethnicity (Han, minority), education (primary school and below, junior high school, senior high school, college or higher) and occupation (farmer, worker, others). Reproductive characteristics included first gestation (yes or no), primipara (yes or no), history of preterm birth (yes or no), history of spontaneous abortion (yes or no), history of induced abortion (yes or no) and history of stillbirth (yes or no). Health status includes BMI (< 18.5 kg/m^2^, 18.5–23.9 kg/m^2^, 24.0–27.9 kg/m^2^ or ≥ 28.0 kg/m^2^), HBsAg (positive or negative), and anemia (yes or no). Living habits during pregnancy include eating meat and eggs (yes or no), no eating vegetables (yes or no), smoking (yes or no), drinking alcohol (yes or no) and taking folic acid (yes or no).

### Statistical analysis

We included all women who fitted the inclusion criteria and described the distributions of conception season in participants with different demographic characteristics, reproductive characteristics, health status and living habits during pregnancy. The *χ*^2^ test was used for inter-group comparison.

Our study analyzed the association between season of conception, month of conception and preterm birth in women of childbearing age, and used multivariate log-binomial model to adjust the potential risk factors for preterm birth and obtained adjusted risk ratio (aRR) and 95% confidence intervals (95% CI) for conception season, conception month and preterm birth. To test the robustness of the results, we adjusted different covariates in stages. In model A, we adjusted for demographic characteristics of participants. In model B, in addition to those factors included in model A, the reproductive characteristics were also adjusted. In model C, in addition to those factors included in model B, we also adjusted for health status of participants. In model D, we additionally adjusted for living habits during pregnancy. All of the analyses were done with SPSS 21 and R 3.6.0 software. Two-sided p values of less than 0.05 was considered statistically significant.

### Patient and public involvement

Participants were not involved in setting the research question or the outcome measures, nor were they involved in the design or implementation of the study. No participants were asked to advise on interpretation or writing up of the manuscript.

## Results

Based on inclusion and exclusion criteria, a total of 209 513 women aged 18–49 years who participated in NFPHEP and had a childbirth outcome from January 1, 2010 to December 31, 2018 were involved in this study. We excluded 8916 with missing information on the last menstrual period; 1633 with missing information on the gestational period; 1756 who had a miscarriage, induced labor or stillbirth and 3126 who had post-term or multiple births. Finally, 194,082 women were included in the study.

**Fig. 2:**
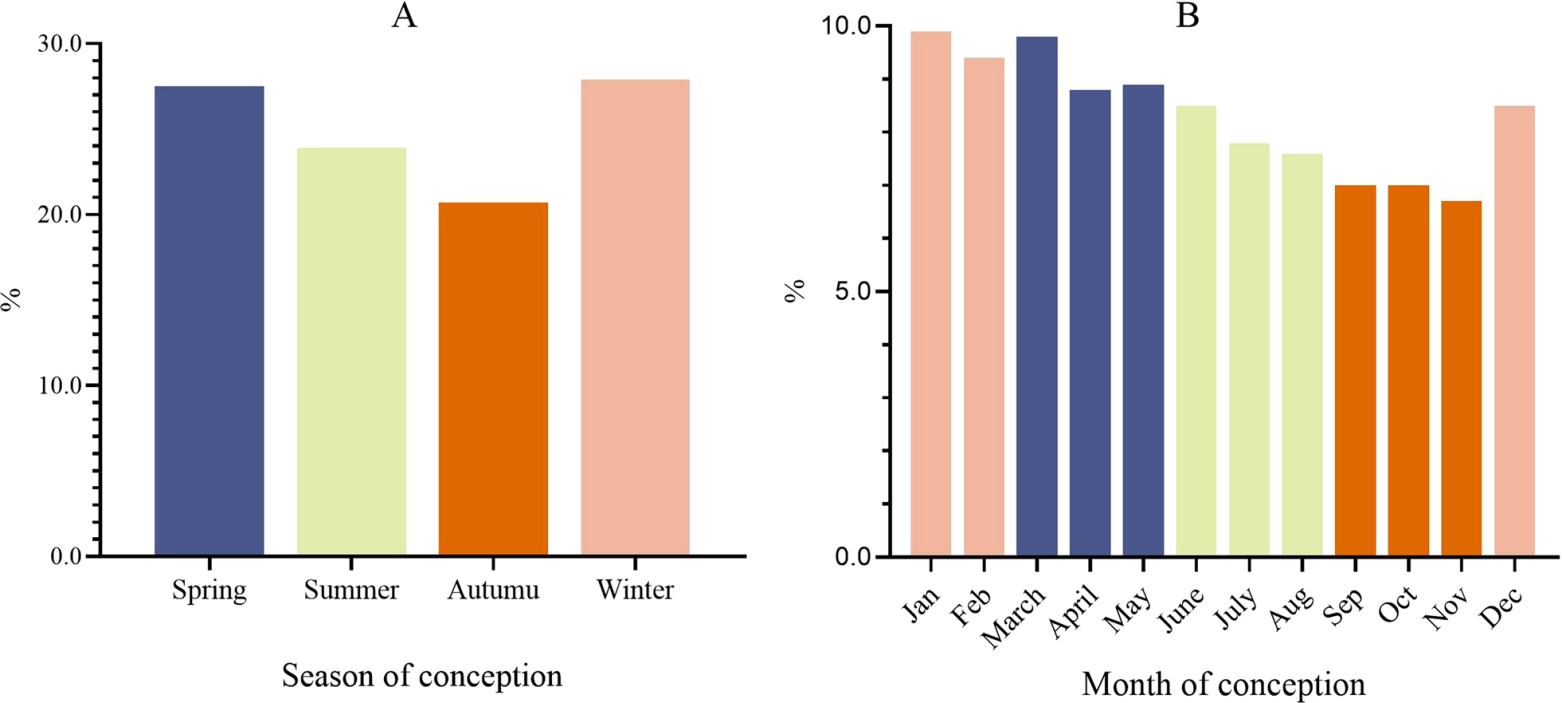
The proportion of women in different season of conception (**A**) and month of conception (**B**)

Of the 194 082 women of childbearing age, 53 334 (27.5%) were pregnant in spring, 46 400 (23.9%) in summer, 40 246 (20.7%) in autumn, and 54 102 (27.9%) in winter (Fig. 2).

The conception season of women with different demographic characteristics (age, ethnicity, education and occupation), reproductive characteristics (gravidity, parity, history of preterm birth, history of spontaneous abortion, history of induced abortion and history of stillbirth), health status (BMI, HBsAg and anemia) and living habits during pregnancy (eating meat and eggs, no eating vegetables, smoking, drinking alcohol and taking folic acid) were all concentrated in spring and winter (Table 1).

**Table 1:** Distribution of the conception season in women with different characteristics

Characteristics	Total	Season of conception			
		Spring	Summer	Autumn	Winter
Demographic characteristics					
Age (years)					
18–20	15,541 (100.0)	4075 (26.2)	3978 (25.6)	3524 (22.7)	3964 (25.5)
21–25	78,227 (100.0)	21,730 (27.8)	17,954 (23.0)	15,821 (20.2)	22,722 (29.0)
26–30	67,951 (100.0)	18,575 (27.3)	16,354 (24.1)	14,317 (21.1)	18,705 (27.5)
31–35	23,515 (100.0)	6477 (27.5)	5856 (24.9)	4786 (20.4)	6396 (27.2)
36–49	8848 (100.0)	2477 (28.0)	2258 (25.5)	1798 (20.3)	2315 (26.2)
Ethnicity*					
Han	120,309 (100.0)	33,463 (27.8)	28,428 (23.6)	24,822 (20.6)	33,596 (27.9)
Minority	73,497 (100.0)	19,803 (26.9)	17,918 (24.4)	15,359 (20.9)	20,417 (27.8)
Education*					
Primary school or below	40,113 (100.0)	10,664 (26.6)	10,057 (25.1)	8569 (21.4)	10,823 (27.0)
Junior high school	104,967 (100.0)	29,087 (27.7)	24,796 (23.6)	21,860 (20.8)	29,224 (27.8)
Senior high school	26,219 (100.0)	7323 (27.9)	6049 (23.1)	5175 (19.7)	7672 (29.3)
College or higher	20,309 (100.0)	5578 (27.5)	4945 (24.3)	4066 (20.0)	5720 (28.2)
Occupation*					
Farmer	178,213 (100.0)	48,938 (27.5)	42,497 (23.8)	36,993 (20.8)	49,782 (27.9)
Worker	8846 (100.0)	2483 (28.1)	2212 (25.0)	1777 (20.1)	2374 (26.8)
Other	5147 (100.0)	1385 (26.9)	1265 (24.6)	1056 (20.5)	1441 (28.0)
Reproductive characteristics					
First gestation*	75,615 (100.0)	21,696 (28.7)	17,094 (22.6)	14,434 (19.1)	22,391 (29.6)
Primipara	93,979 (100.0)	26,820 (28.5)	21,265 (22.6)	18,014(19.2)	27,880 (29.7)
History of preterm birth	731 (100.0)	184 (25.2)	178 (24.4)	161(22.0)	208 (28.5)
History of spontaneous abortion	7063 (100.0)	1845 (26.1)	1733 (24.5)	1584(22.4)	1901(26.9)
History of induced abortion	45,628 (100.0)	12,288 (26.9)	10,851 (23.8)	9531 (20.9)	12,958 (28.4)
History of stillbirth	1696 (100.0)	405 (23.9)	422 (24.9)	373 (22.0)	496 (29.2)
Health status					
Body-mass index (kg/m2)*					
< 18.5	23,513 (100.0)	6491 (27.6)	5564 (23.7)	4783 (20.3)	6675 (28.4)
18.5–23.9	137,343 (100.0)	37,779 (27.5)	32,798 (23.9)	28,454 (20.7)	38,312 (27.9)
24.0–27.9	24,520 (100.0)	6619 (27.0)	5965 (24.3)	5207 (21.2)	6729 (27.4)
≥ 28.0	5477 (100.0)	1519 (27.7)	1309 (23.9)	1168 (21.3)	1481 (27.0)
Anemia*	46,043 (100.0)	12,367 (26.9)	10,989 (23.9)	9561 (20.8)	13,126 (28.5)
HBsAg positive*	4767 (100.0)	1283 (26.9)	1158 (24.3)	1030 (21.6)	1296 (27.2)
Living habits during pregnancy					
Eating meat and eggs	192,443 (100.0)	52,872 (27.5)	46,025 (23.9)	39,924 (20.7)	53,622 (27.9)
No eating vegetables	765 (100.0)	215 (28.1)	203 (26.5)	161 (21.0)	186 (24.3)
Smoking*	710 (100.0)	199 (28.0)	179 (25.2)	148 (20.8)	184 (25.9)
Drinking alcohol*	4376 (100.0)	1156 (26.4)	1080 (24.7)	974 (22.3)	1166 (26.6)
Taking folic acid*	184,547 (100.0)	50,775 (27.5)	44,001 (23.8)	38,152 (20.7)	51,619 (28.0)

*Denominators provided were some data were missing

**Fig. 3:**
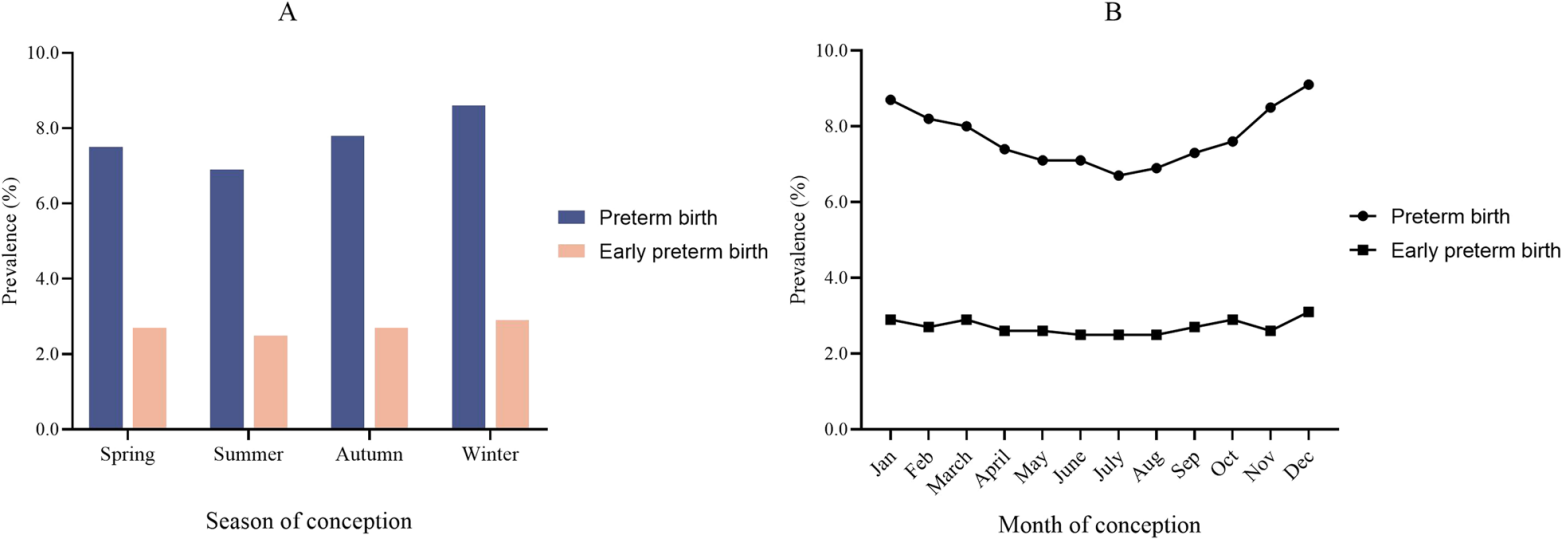
Preterm birth rate and early preterm birth rate of pregnancies with different season of conception (**A**) and month of conception (**B**)

The average gestational week of 194082 women was 39.37 ± 2.23 weeks, of which 15034 women had preterm birth, accounting for 7.7% of the total participants, and 5287 women showed early preterm birth, accounting for 2.7% of the total participants. The preterm birth rates of pregnancies in spring, summer, autumn and winter were 7.5%, 6.9%, 7.8%, and 8.6%, respectively, and the early preterm birth rates were 2.7%, 2.5%, 2.7%, and 2.9%, respectively (Fig. 3). The difference was statistically significant (P < 0.05).

The preterm birth rate and early preterm birth rate of pregnancies that were conceived from January to December showed a U-shaped trend (Fig. 3). Among them, the preterm birth rate (6.7%) and early preterm birth rate (2.5%) of pregnancies in July were the lowest, and the preterm birth rate (9.1%) and the early preterm birth rate (3.1%) of pregnancies in December were the highest, and the difference was statistically significant (P < 0.05).

Univariate log-binomial regression model showed that the season of conception was related to the preterm birth and early preterm birth (P < 0.05). Compared with pregnancies that were conceived in the summer, pregnancies that were conceived in the spring, autumn and winter had the higher risk of preterm birth (Spring: RR = 1.10, 95% CI: 1.04 to 1.15; Autumn: RR = 1.14, 95% CI: 1.09 to 1.20; Winter: RR = 1.28, 95% CI: 1.22 to 1.34) and also had a higher risk of early preterm birth (Spring: RR = 1.09, 95% CI: 1.01 to 1.18; Autumn: RR = 1.09, 95% CI: 1.01 to 1.19; Winter: RR = 1.16, 95% CI: 1.08 to 1.25). Multivariate log-binomial model showed that after adjusting for covariates, compared with pregnancies that were conceived in summer, pregnancies in spring, autumn and winter were at increased risk of preterm birth by 10%, 15%, and 28%, respectively, and at increased risk of early preterm birth by 9%, 10%, and 18%, respectively. The adjustment of different covariables did not affect the results, and the results of the four models were robust (Table 2).

**Table 2:** Association between the season of conception with preterm birth and early preterm birth after adjusting for different covariates

	Season of conception			
Outcome	Spring	Summer	Autumn	Winter
Preterm birth				
Crude RR (95%CI)	1.10 (1.04,1.15)	1.00	1.14 (1.09,1.20)	1.28 (1.22,1.34)
Adjusted RR (95%CI)*				
Model A	1.10 (1.05,1.15)	1.00	1.14 (1.08,1.20)	1.28 (1.22,1.34)
Model B	1.10 (1.05,1.15)	1.00	1.14 (1.08,1.20)	1.28 (1.22,1.34)
Model C	1.10 (1.05,1.16)	1.00	1.14 (1.08,1.20)	1.28 (1.22,1.35)
Model D	1.10 (1.05,1.16)	1.00	1.15 (1.09,1.21)	1.28 (1.22,1.34)
Early preterm birth				
Crude OR (95%CI)	1.09 (1.01,1.18)	1.00	1.09 (1.01,1.19)	1.16 (1.08,1.25)
Adjusted OR (95%CI)*				
Model A	1.09 (1.01,1.18)	1.00	1.09 (1.01,1.19)	1.16 (1.08,1.25)
Model B	1.09 (1.01,1.18)	1.00	1.09 (1.01,1.19)	1.16 (1.08,1.25)
Model C	1.09 (1.01,1.18)	1.00	1.10 (1.01,1.19)	1.17 (1.09,1.26)
Model D	1.09 (1.01,1.18)	1.00	1.10 (1.00,1.19)	1.18 (1.09,1.27)

*Model A adjusted for sociodemographic characteristics (age, ethnicity, education level, occupation);Model B adjusted for those factors included in model A and reproductive characteristics (first gestation, primipara, history of preterm birth, history of spontaneous abortion, history of induced abortion, history of stillbirth);Model C adjusted for those factors included in model B and health status (BMI, anemia, HBsAg);Model D adjusted for those factors included in model C and living habits during pregnancy (eating meat and eggs, no eating vegetables, smoking, drinking alcohol, taking folic acid)

**Fig. 4:**
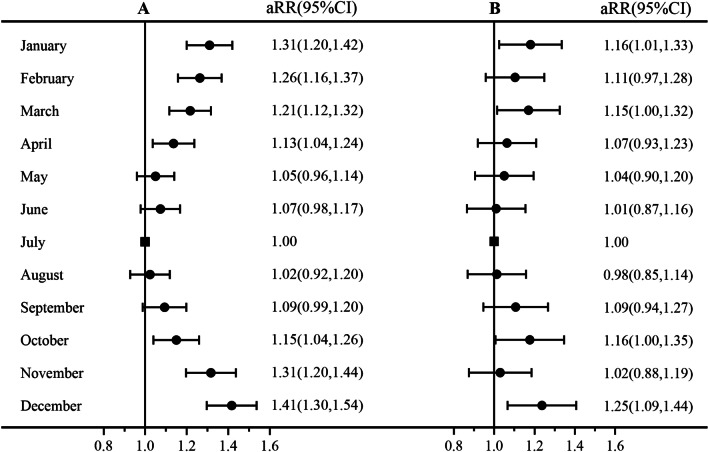
Association between month of conception with preterm birth (**A**) and early preterm birth (**B**). *Adjusted for demographic characteristics (age, ethnicity, education level and occupation), reproductive characteristics (first gestation, primipara, history of preterm birth, history of spontaneous abortion, history of induced abortion and history of stillbirth), health status (BMI, anemia and HBsAg) and living habits during pregnancy (eating meat and eggs, no eating vegetables, smoking, drinking alcohol and taking folic acid)

Multivariate log-binomial model showed that after adjusting for demographic characteristics, reproductive characteristics, health status and living habits during pregnancy, compared with pregnancies that were conceived in July, pregnancies in November, December, and January had a higher risk of preterm birth, with aRR values of 1.31 (95% CI: 1.20 to 1.44), 1.41 (95% CI: 1.30 to 1.54), and 1.31 (95% CI: 1.20 to 1.42), respectively; pregnancies in October, December, and January had a higher risk of early preterm birth, with aRR values of 1.16 (95% CI: 1.00 to 1.35)、1.25 (95% CI: 1.09 to 1.44) and 1.16(95% CI: 1.01 to 1.33), respectively (Fig. 4).

## Discussion

Our study results revealed that preterm and early preterm birth were significantly related to the season of conception. Among the women of childbearing age in southwest China, the preterm and early preterm birth rates were the highest in pregnancies conceived in winter, and lowest in pregnancies conceived in summer. The risk of preterm birth increased by 10%, 15%, and 28%, and that of early preterm birth increased by 9%, 10%, and 18% in pregnancies conceived in spring, autumn, and winter, respectively, compared with those conceived in summer. Our study demonstrated that the preterm and early preterm birth rates of pregnancies conceived from January to December exhibited a U-shaped trend. The preterm birth rate (6.7%) and early preterm birth rate (2.5%) of pregnancies conceived in July were the lowest, and the preterm birth rate (9.1%) and the early preterm birth rate (3.1%) of pregnancies conceived in December were the highest; this finding is consistent with seasonal f preterm birth.

Studies examining the association of the season of conception and the risk of preterm birth are limited. Previous studies conducted in the United Kingdom [15]; Greece [17]; and Guangdong, China [22] have all examined the association among birth month, birth season and preterm birth. However, birth month may be identical for two children with different gestational ages who may have been conceived at different times and have different in utero exposures. Therefore, the month of conception may be a more sensitive marker for in utero exposure compared with the month of birth [23]. A retrospective cohort study conducted in Pennsylvania, the United States, revealed that pregnancies conceived in the summer and autumn had the lowest prevalence of preterm birth < 37 weeks (P < 0.01), spontaneous preterm birth at < 37 weeks (P < 0.01), preterm birth at < 32 weeks (P < 0.01) and spontaneous preterm birth at < 32 weeks from 1 to 1995 to 31 December 2005 [24]. After adjustment for maternal age, race/ethnicity, marital status, education level and parity, pregnancies conceived in summer and autumn exhibited a reduction of 8–19% in the risk of subtypes of preterm birth [24]. In their study conducted in the Germany, Wolf et al. [25] chose Brandenburg and Saxony, two states with very similar average temperatures (mean temperature ranged in Brandenburg between 9.1 and 9.6 °C and in Saxony between 9.0 and 9.7 °C), and determined that the risk of preterm birth was slightly higher in pregnancies conceived in spring than in those conceived in summer in Brandenburg but not in Saxony. The level of evidence considerably differed between the two states. However, in their study performed in North Carolina, the United States, Miranda et al. [26] observed that the prevalence of preterm birth was higher in pregnancies conceived in spring (OR = 1.10, 95% CI: 1.05 to 1.16) and summer (OR = 1.06, 95% CI: 1.01 to 1.12) than in those conceived in winter. Seasonal patterns were the most pronounced among non-Hispanic white women living in urban areas.

In their retrospective cohort conducted in the United States, Bodnar et al. [24] observed that the prevalence of preterm birth < 37 weeks was the highest in pregnancies conceived in March and the lowest in September. Furthermore, the prevalence of preterm birth at < 32 weeks was the highest in pregnancies conceived in March and May and the lowest in pregnancies conceived in August. Based on the logistic regression model fitting the first pair of Fourier series terms, the average peak prevalence of preterm birth < 37 weeks, preterm birth < 32 weeks and spontaneous preterm birth < 32 weeks were observed among early spring conceptions and had an average nadir among early autumn conceptions; the average peak prevalence of spontaneous preterm birth < 37 weeks was observed found among late winter conceptions and had an average nadir among summer conceptions. Weinberg et al. [27] conducted a study in Norway covering 2,321,652 births and observed that the peak prevalence of preterm birth occurred near early January and early July. The seasonal variation based on fetuses revealed two peaks for early preterm, coinciding with New Year’s Day and the early July beginning of Norway’s summer break, and may reflect a holiday-related pattern of unintended conception. Countries differ in the climatic characteristics of their seasons; thus, the effect of conception month on preterm birth is not uniform across countries.

Conflicting or slightly varying results between countries were most likely related to geographical, cultural and socio-economic differences between the study populations. Globally, the preterm birth rate showed different seasonal trends in different regions. For example, in high-latitude countries such as the United Kingdom and Sweden, the rate of preterm birth was higher in winter, [15, 28], while in low-latitude countries such as Greece and Japan, the rate of preterm birth was higher in spring and summer [16, 28], indicating that the seasonal pattern of preterm birth may be related to latitude. Countries with different latitudes have different climate characteristics. Certain environmental factors (such as temperature, air pressure, air pollution, light) are related to the seasons, leading to seasonal variations in preterm birth. In low socioeconomic areas, traffic-related air pollution is linked to preterm birth risk, especially in winter. Liu et al. [29] found that the concentrations of air pollutant (PM2.5, PM10, SO2, NO2 and CO) had obvious seasonal trends with the highest in winter and the lowest in summer. After controlling for the impact of confounding factors, the increases in the risk of preterm birth were associated with each 10 g/m^3^ increase in PM_2.5_ (OR = 1.043, 95% CI: 1.01 to 1.09) and PM_10_ (OR = 1.039, 95% CI: 1.01 to 1.14) during the first trimester. Regarding preterm birth, mechanisms that implicate toxic fine particulates include maternal hematologic transport of inhaled noxious chemicals, the triggering of systemic inflammation, or alterations in function of the autonomic nervous system [30]. Ultraviolet sunlight exposure and maternal vitamin D status may also be relevant to the seasonality of preterm birth. Sunlight is the major contributor to vitamin D status. Because of the seasonal variation in ultraviolet light, vitamin D nutritional status is best in summer and autumn and poorest in winter and spring [24]. A review in 2019 revealed that vitamin D deficiency may contribute to the pathophysiology of preterm birth. The main mechanism associated with vitamin D’s role in preventing preterm birth is likely its effect on the innate immune response [31]. Vitamin D receptors are present in immune cells such as macrophages and dendritic cells that recognize molecules derived from microbes. Once activated, these immune cells produce antimicrobial peptides. This antimicrobial pathway may be instrumental in preventing perinatal infections that are associated with preterm birth [31]. Previous studies had confirmed that infection was one of the main causes of preterm birth, and infection in the first trimester was closely related to preterm birth [32]. Some reproductive tract infections (gonorrhea, chlamydia trachomatis, etc.) also revealed a certain seasonality [33–35], which can explain the seasonality of preterm birth to a certain extent.

Nutritional status during pregnancy may also affect the preterm birth rate [36]. Previous studies revealed that better maternal diet quality during pregnancy, characterized by a high intake of vegetables, fruits, whole grains, dairy products and protein diets, may reduce the risk of preterm birth [37]. Seasonal patterns in nutritional status and maternal weight loss have been implicated as factors responsible for the seasonal pattern of low birthweight in developing countries. However, these factors are unlikely to play a large role in developed countries [38, 39]. In addition, some studies had found that the seasonal pattern of conception may be associated with sociodemographic characteristics (age, occupation, education level, marital status, etc.), and the risk of preterm birth was not consistent among women with different sociodemographic characteristics, thus explaining the seasonality of preterm birth to a certain extent [40, 41].

Previous studies had found that season of conception was also associated with other adverse pregnancy outcomes. A study by Benavides et al. [23] in Texas, USA revealed that season of conception was associated with 5% of birth defects. Offspring conceived in the summer had a higher incidence of birth defects, particularly congenital megacolon disease and other congenital colonic functional disorders. There were significantly increased prevalence ratios for any monitored birth defect among conceptions in May, June, July, August, and September, relative to January. Conception in March was negatively associated with prevalence of any birth defect compared to January. A study by Hebert et al. [42] in the UK showed that the risk of autism spectrum disorder for children conceived in spring, summer and winter was 1.10 (95% CI: 0.54 to 2.24), 2.08 (95% CI: 1.18 to 3.70), and 1.25 (95% CI: 0.54 to 2.24) times that of children conceived in autumn, which suggested a higher proportion of children with autistic spectrum disorder being conceived in the summer.

From a biological perspective, there is a critical period spanning the weeks around conception when gametes mature, fertilization occurs and the developing embryo forms. These are the events most sensitive to environmental factors such as the availability of macro- and micronutrients or exposure to smoking, alcohol, drugs or other teratogens [36]. Therefore, compared with the birth season, the conception season is more important to the health of the offspring. Investigating the seasonality of preterm birth can provide new insights useful in limiting the risk of preterm birth. Ecological association studies such as this provide a guide to more definitive studies in the search for causal factors in adverse birth outcomes. One way to evaluate these potential seasonal risk factors would be to intervene and to assess whether a specific intervention reduces the seasonality of the adverse birth outcomes. The expected uptake in antenatal visits is a good opportunity to intervene on nutritional status during pregnancy and to treat women for various morbidities that might reduce the prevalence of adverse birth outcomes. Maternal influenza infections could be reduced with a vaccination program. Improving the nutritional status of women who are pregnant in winter and preparing for fetuses with higher risk of preterm birth will help reduce the preterm birth rate in Southwest China. Further studies are required to confirm these findings and identify specific environmental or other factors that may drive seasonal patterns.

Our study has several strengths. First, this study was a large population-based retrospective cohort study that recruited all women who participated in the NFPHEP and had a singleton livebirth from 129 counties of Southwest China between 2010 and 2018, which may have less selection bias. Second, our study adjusted the demographic characteristics, reproductive characteristics, health status and living habits during pregnancy which were related to preterm birth in stages, especially the HBsAg positive [4] and folic acid supplementation [43] found in the previous study that may increase or reduce the risk of preterm birth, and the results of the association between conception season and preterm birth were more reliable. In addition, this was the first study to explore the association between season of conception, month of conception and preterm birth in Southwest China. The cohort included more than 40 ethnic minorities living in the region, and our results are more representative of the multi-ethnic situation in China than other studies.

Our study has some limitations. The NFPHEP did not collect information on pregnancy complications such as gestational hypertension, gestational diabetes, and therefore may influence the interpretation of the results to some extent. Although we have found the seasonal characteristics of preterm birth, we cannot determine the mechanism of the conception season’s influence on preterm birth. Further research is still needed for in-depth exploration.

## Conclusions

Preterm birth exhibited a seasonal variation, and preterm and early preterm birth were significantly related to season of conception. Preterm and early preterm birth rates were the highest among pregnancies conceived in winter, and preterm and early preterm birth rates were the lowest among pregnancies conceived in summer. This trend should raise concern since prematurity places a huge toll emotionally, physically and financially on families, medical systems and regional or national economies. Improved identification of the causes for rising rates of preterm birth in China and appropriate management to minimize untimely birth remains a challenge.

## Data Availability

The datasets generated and/or analysed during the current study are not publicly available because the data contains personal information but are available from the corresponding author on reasonable request.
